# PoopMD, a Mobile Health Application, Accurately Identifies Infant Acholic Stools

**DOI:** 10.1371/journal.pone.0132270

**Published:** 2015-07-29

**Authors:** Amy Franciscovich, Dhananjay Vaidya, Joe Doyle, Josh Bolinger, Montserrat Capdevila, Marcus Rice, Leslie Hancock, Tanya Mahr, Douglas B. Mogul

**Affiliations:** 1 Department of Pediatrics, Johns Hopkins University School of Medicine, Baltimore, Maryland, United States of America; 2 Department of Epidemiology, Johns Hopkins University Bloomberg School of Public Health, Baltimore, Maryland, United States of America; 3 HCB Health, Austin, Texas, United States of America; 4 Office of Technology Transfer, Johns Hopkins University, Baltimore, Maryland, United States of America; University of Erlangen-Nuremberg, GERMANY

## Abstract

Biliary atresia (BA) is the leading cause of pediatric end-stage liver disease in the United States. Education of parents in the perinatal period with stool cards depicting acholic and normal stools has been associated with improved time-to-diagnosis and survival in BA. PoopMD is a mobile application that utilizes a smartphone’s camera and color recognition software to analyze an infant’s stool and determine if additional follow-up is indicated. PoopMD was developed using custom HTML5/CSS3 and wrapped to work on iOS and Android platforms. In order to define the gold standard regarding stool color, seven pediatricians were asked to review 45 photographs of infant stool and rate them as acholic, normal, or indeterminate. Samples for which 6+ pediatricians demonstrated agreement defined the gold standard, and only these samples were included in the analysis. Accuracy of PoopMD was assessed using an iPhone 5s with incandescent lighting. Variability in analysis of stool photographs as acholic versus normal with intermediate rating weighted as 50% agreement (kappa) was compared between three laypeople and one expert user. Variability in output was also assessed between an iPhone 5s and a Samsung Galaxy S4, as well as between incandescent lighting and compact fluorescent lighting. Six-plus pediatricians agreed on 27 normal and 7 acholic photographs; no photographs were defined as indeterminate. The sensitivity was 7/7 (100%). The specificity was 24/27 (89%) with 3/27 labeled as indeterminate; no photos of normal stool were labeled as acholic. The Laplace-smoothed positive likelihood ratio was 6.44 (95% CI 2.52 to 16.48) and the negative likelihood ratio was 0.13 (95% CI 0.02 to 0.83). kappa_user_ was 0.68, kappa_phone_ was 0.88, and kappa_light_ was 0.81. Therefore, in this pilot study, PoopMD accurately differentiates acholic from normal color with substantial agreement across users, and almost perfect agreement across two popular smartphones and ambient light settings. PoopMD may be a valuable tool to help parents identify acholic stools in the perinatal period, and provide guidance as to whether additional evaluation with their pediatrician is indicated. PoopMD may improve outcomes for children with BA.

## Introduction

Biliary atresia (BA) is a perinatal disease of hepatobiliary destruction that causes cirrhosis and is universally fatal without intervention. BA is the most common cause of neonatal cholestasis and accounts for nearly half of all pediatric liver transplants in the United States [1]. Significant morbidity and mortality result from consequences of chronic and end-stage liver disease (ESLD) including malnutrition and growth failure, cognitive impairments, and gastrointestinal bleeding from portal hypertension. Earlier time-to-diagnosis and subsequent surgical intervention with a hepatoportoenterostomy (HPE or “Kasai” procedure) is associated with better outcomes, including an increase in overall survival and decrease in rates of transplantation. The best outcomes occur in infants who are diagnosed and undergo HPE within 60 days of life [2–5]. However, in the United States, diagnosis of BA is typically delayed beyond the optimal timepoint for intervention [6]. Because physiologic jaundice of the newborn is extremely common and mimics the most common sign of BA, icterus, recognizing that an infant has BA proves challenging for health care providers. Adding to the difficulty of diagnosis is limited awareness for BA on the part of both parents and health care providers since it is a rare disease with an estimated incidence of 1 in 13,000 in the United States, or approximately 400 new cases per year [7].

One potential clue to help distinguish BA from physiologic jaundice is that babies with BA often have pale (i.e., acholic) stools. Consequently, Taiwan implemented the first national stool color card screening program and data registry in 2004 [8]. This program provides parental education through the distribution of a stool card that has pictures of pale and normal stool as well as providing extensive physician education. Implementation of this program corresponded with significant improvement in time-to-diagnosis, increase in overall survival and jaundice-free survival, as well as a marked reduction in the rate of liver transplantation [9,3].

The use of mobile applications (“apps”) provides an additional opportunity to educate parents of newborns regarding the clinical significance of acholic stools and the value in early detection for cholestatic liver diseases such as BA. Over the last few years, health-related mobile app development and use has increased significantly due to recognition of the potential for both far-reaching societal impacts as well as financial gains in this field. Fifty-six percent of American adults own a smartphone, including ~80% of individuals 18–35 years of age, and use within this age group is largely independent of income [10]. Among smartphone users, 31% use their phone to look up health information and 20% use a health app [11]. Consequently, we developed PoopMD, a free mobile application that utilizes a smartphone’s camera and color recognition software to analyze an infant’s stool and educate parents about normal and abnormal stool color in neonates. This app can then alert the user whether additional follow-up is indicated. In this pilot study, we tested the accuracy of PoopMD in differentiating photographs of acholic and normal stools.

## Materials and Methods

### Software Development

In collaboration with HCB Health (Austin, TX), PoopMD was developed using custom HTML5/CSS3 code and wrapped to work on iOS and Android platforms. Conceptual framework, artistic design, components of the user interface, functionality and navigability were discussed amongst team members. Original designs were revised through an iterative process using dynamic feedback via task-based user testing. Version 1 of the application was made available in the Apple “App Store” and Google Play in April 2014.

### Color Analyzer

**Fig 1:**
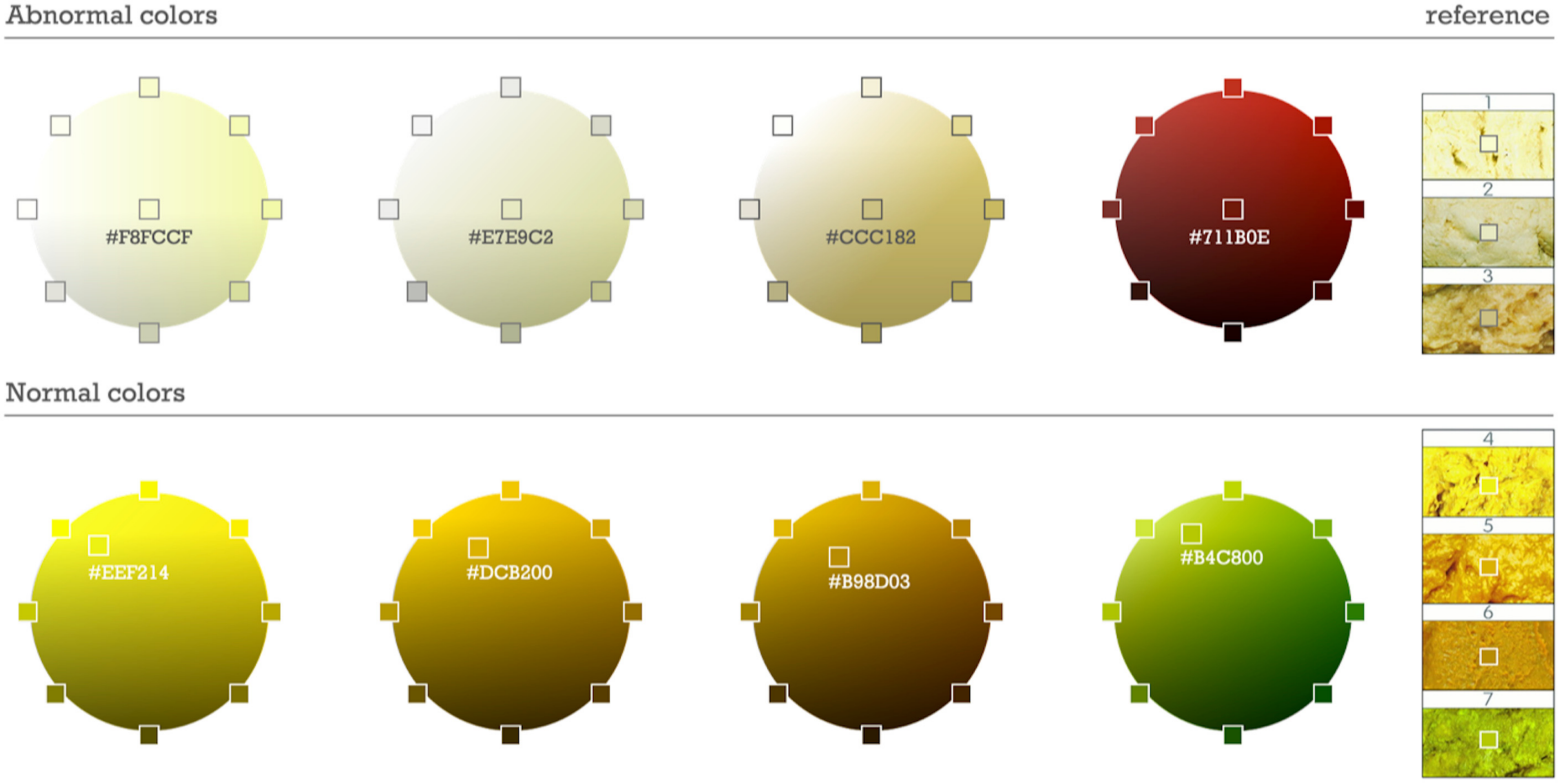
RGB hexcodes denoting the spectrum of abnormal and normal stool colors.

Digital cameras with color recognition software are built within the originally manufactured mobile smartphone and are able to utilize customized software such as mobile apps. Colors from previously-validated images of normal and acholic stool from the Taiwan Stool Card were converted to a 16-base color code using Color-hex, an open source color library (see www.color-hex.com; Fig 1). Acholic was defined as a range from white to the pale color hexes captured from the acholic stools on the Taiwan stool card, and accounted for variations in hue and brightness [9]. Normal stool colors were selected in a similar way, and were refined with testing from a collection of over 100 photos of stools considered normal by a pediatric gastroenterologist (DBM). Finally, analysis of stool color suggestive of gastrointestinal bleeding was developed using photographs of “bright red blood per rectum” and the color spectrum that exists between these color hexes and black.

### Navigating PoopMD

**Fig 2:**
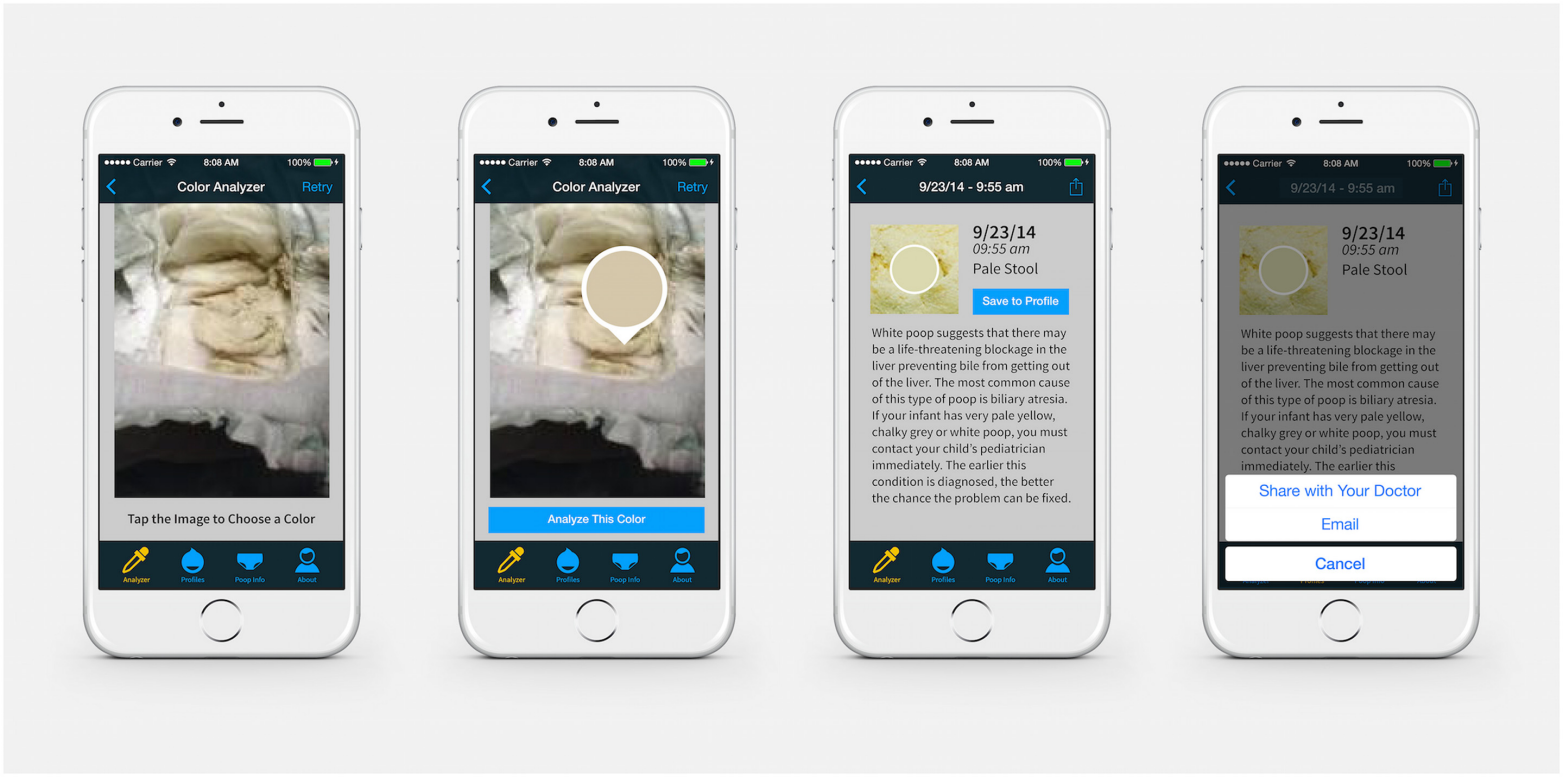
Screenshots demonstrating PoopMD analytic functionality.

PoopMD was designed with an intuitive user interface, with the target audience being new parents inclusive of late adolescents and young adults, assuming an 8^th^ grade reading proficiency. Serial prompts with touch screen buttons to select appropriate actions are demonstrated in Fig 2. The user may select a photo from “Photo Library” or capture a new image with the camera. Subsequently, the user will select with a “dropper” tool the area of concern within the stool. PoopMD then analyzes the color as normal, pale, or consistent with blood. If the color selected is unable to match the color hexes assigned for rendering these three results, the result will be labeled “indeterminate,” and the user will be prompted to select a new submission or take a new photograph with tips to enhance quality. Images may be saved to a specific baby’s profile for future viewing.

### Establishing the Gold Standard

**Fig 3:**
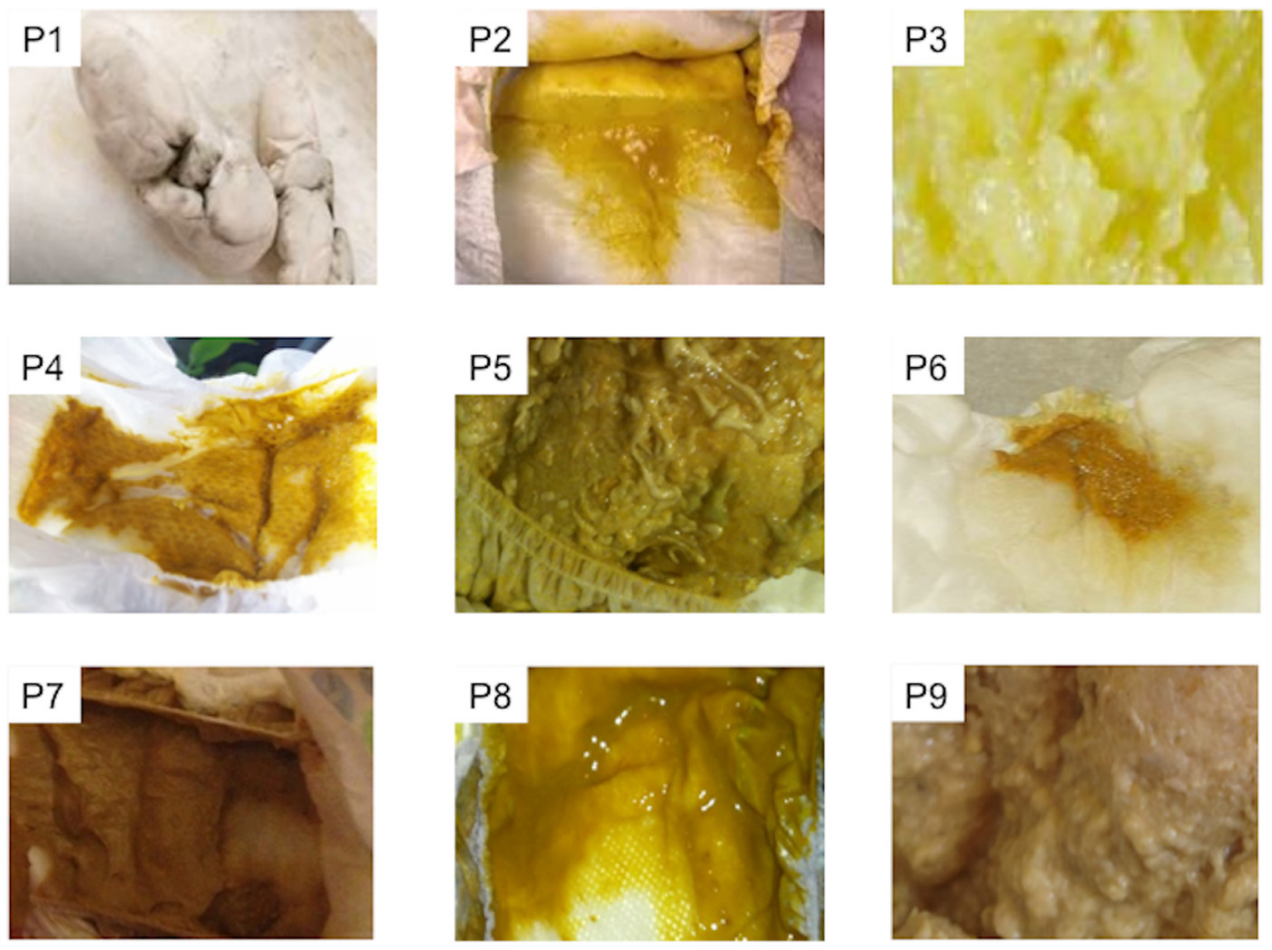
Example of stool photographs presented to panel of pediatricians for development of gold standard.

In order to define the gold standard for assessment of normalcy of stool color, a panel of seven expert pediatricians comprised of general practitioners, gastroenterologists and emergency medicine physicians were asked to review 45 photographs of infant stool and rate them as acholic, normal, or indeterminate. Since PoopMD was developed with the primary objective to help parents better understand the importance of acholic stools, and because parents are likely to recognize red or black stools as suggestive of potential gastrointestinal bleeding, images of bloody stools were excluded from this analysis (Fig 3). Photographs of stool samples were collected from volunteers and online resources without associated protected health information (PHI) and no PHI was visible to the physicians. Only stools for which 6+ pediatricians were in agreement—normal, acholic or indeterminate—were included in the analysis.

### Assessing Device Accuracy

Initial conditions for testing the accuracy of PoopMD included use of an iPhone 5s under incandescent lighting without a flash by an expert user (DBM). The response generated by PoopMD was recorded for each photo as “acholic” or “normal.” If PoopMD was not able to resolve the color, stating the color as “indeterminate” on three successive attempts, the result was recorded as indeterminate. Sensitivity (i.e., percentage of true acholic stools that PoopMD analyzed as acholic) and specificity (i.e., the percentage of true normal stools that PoopMD analyzed as normal) were determined. The positive and negative likelihood ratios for the test were calculated per Deeks and Altman, as sensitivity/(1—specificity), and (1—sensitivity)/specificity, respectively [12]. Because 100% sensitivity is not compatible with the calculation of negative likelihood ratio, we used Laplace smoothing for the calculation of the negative likelihood ratio. Laplace smoothed sensitivity is equal to (true positives + 1)/(number with disease + 2); Laplace smoothed specificity is equal to (true negatives + 1)/(number without disease + 2). Laplace smoothing provides conservative estimates of the likelihood ratios given small numbers. Variability in PoopMD analysis was assessed with intermediate rating weighted as 50% agreement, and was determined between three laypeople (kappa_user_), with the images imported into the iPhone photo stream. Variability in output was also determined between an iPhone 5s and a Samsung Galaxy S4 kappa_phone_), as well as between incandescent lighting and compact fluorescent lighting kappa_light_. By convention, a kappa between 0.60 and 0.79 was defined as moderate agreement and a kappa between 0.80 and 1 was defined as substantial agreement.

### Ethics Statement

This study involved the use of de-identified photographs and was therefore approved by the Johns Hopkins Medicine Institutional Review Board for an IRB exemption given that it does not involve human subjects research under the DHHS or FDA regulations.

## Results

Among the initial 45 photographs, there was agreement between 6+ pediatricians on 34 samples including 27 photographs that were defined as true normal stools and 7 photographs that were defined as true acholic stools; there was no consensus among pediatricians that any photographs represented an indeterminate stool. These 34 photographs became the gold standard set of images. The sensitivity of PoopMD under initial conditions was 100% (7 of 7) with no false negatives (Table 1). The specificity of PoopMD under initial conditions was 89% (24 of 27) with 3 images (11%) falsely labeled as indeterminate and no images falsely labeled as acholic.

**Table 1:** Accuracy of PoopMD.

		Gold standard		
		Acholic	Normal	Indeterminate
PoopMD	Acholic	7	0	0
	Normal	0	24	0
	Indeterminate	0	3	0
		7	27	0

The Laplace-smoothed positive likelihood ratio for the test is 6.44 (95% CI 2.52 to 16.48) and the Laplace-smoothed negative likelihood ratio was 0.13 (95% CI 0.02 to 0.83). PoopMD was also tested under varying conditions including a range of users, phone types and ambient lighting. The kappa_user_ was 0.68, demonstrating moderate agreement, while the kappa_phone_ was 0.88 and kappa_light_ was 0.81 and consistent with substantial agreement. All raw data has been provided (S1 Table).

## Discussion

This pilot study demonstrates that PoopMD, a free mobile app available in the Apple and Google stores, can accurately identify images of acholic stool. Specifically, PoopMD was able to correctly identify all examples of acholic stools with no false negatives. Although PoopMD misclassified 11% of normal stools as indeterminate, the app never stated that a normal stool was acholic, which would cause unnecessary anxiety for a parent/user. The positive and negative likelihood ratios from our study further support the conclusion that results from PoopMD accurately identify individuals that have acholic stools or normal stools. Furthermore, analysis of these images was consistent across a range of users, smartphones and ambient lighting. Consequently, PoopMD may represent a valid and reliable way to identify acholic stools, a characteristic finding in babies with BA.

Stool color education has been shown to improve survival of children with BA. Specifically, the Taiwanese government has implemented a national BA registry and stool color card program since 2004, where all parents of newborns are given a stool card and asked to notify their pediatrician if their baby’s stool looks like one of the photographs of acholic stools. This intervention has been shown to not only shorten the time to diagnosis, but has led to an increase in the 5-year jaundice-free survival (i.e., “successful Kasai”) from 27% to 64% and an increase in the 5-year overall mortality from 56% to 89% [3]. Furthermore, even in countries such as the United States and Canada where the incidence of BA is slightly lower as compared to east Asian countries, a stool color card education/screening program has been shown to be cost-effective [13,14]. At the same time, evidence suggests that health care providers are often not able to differentiate acholic stools from normal stools [15]. These findings support the need for educational tools that not only depict images of stool with varying color, but also strongly reinforce: (1) the clinical implications of acholic stool and (2) the need to act quickly to determine the underlying etiology should intervention be necessary. Furthermore, earlier identification of acholic stools would facilitate evaluation for other disorders of neonatal cholestasis such as a choledochal cyst or Alagille’s syndrome, and would differentiate these clinically significant etiologies from transient acholic stools that require no additional intervention.

Although stool color cards represent a novel and highly effective tool in improving outcomes in BA, PoopMD offers several potential advances and advantages. First, individuals are using smartphones for an increasing number of hours per day, with some studies suggesting that individuals spend 2–3 hours a day using a smartphone [16,17]. Therefore, a mobile app is a familiar tool that provides an optimal way to educate parents about newborn stool color, and learn if their baby has this rare disease. Moreover, PoopMD educates parents to understand that nearly all stool colors are normal, an observation that can be reinforced with continued use of the color analyzer. PoopMD is advantageous compared to traditional cards because parents can elect to receive automated reminders every two weeks for a total of eight weeks, the critical window for diagnosis of BA. Finally, individuals who possess their physician’s email address can easily send a stool photograph to the pediatrician with an automated message that includes the output from the color analyzer and a PoopMD description of the benefit for early intervention.

Currently, only Taiwan has implemented a nationwide screening program for BA through its stool color card program. However, inclusion of screening for BA as part of the newborn screen may be warranted in the US and elsewhere. Specifically, according to the World Health Organization (WHO), screening is indicated when: (1) the condition is an important public health problem; (2) the natural history is clearly defined; (3) there is a detectable early stage; (4) treatment at an early stage is of greater benefit than at a later stage; (5) a suitable test exists that detects an early stage; (6) the test is acceptable; (7) the risks, both physical and psychological, are less than the benefits; and (8) the costs are balanced against the benefits [18]. In the case of BA, conditions one through four are well-understood to have been met, with a disease that accounts for nearly 50% of pediatric liver transplants, when earlier detection leads to better outcomes, and which the average time to detection is significantly delayed. Whereas stool color cards, direct bilirubin measurements and dried blood spots have all been proposed as possible tests (criteria five through seven) for newborn screening in BA, this study identifies the possibility that a mobile app may provide a novel way to screen for a disease and may satisfy all WHO criteria. Although smartphones are currently owned by only 80% of individuals of child-bearing age, including those individuals with little-to-no income, this incomplete penetrance should not limit the integration of mobile health solutions into the public health system since many screening programs embraced by the American Academy of Pediatrics and its periodicity table are only utilized by a fraction of providers and patients [19,20]. Likewise, the precedent for use of mobile applications for public programs already exists, for example, the Integrated Public Alert and Warning System notifies smartphone owners via text messages when a child has been kidnapped (i.e., Amber Alert) [21].

This pilot study represents an initial assessment of how a mobile app could potentially be used to screen for a disease by aligning results from a biosensor, in this case the camera, with a clinically meaningful sign and physical exam finding. Currently, there are estimated to be over 90,000 mobile apps directed towards healthcare and there is tremendous enthusiasm for the potential of these apps—i.e., the use of mobile health, or mHealth, technologies—to improve health [22]. Applications aim to not only screen for new problems, but also to improve ongoing monitoring of known health problems outside the hospital and clinic including changes in heart rate and rhythm, breathing abnormalities, sleep and nearly every area of health and illness. Though the development of new mobile health applications is increasing at an extremely rapid pace, only a very small fraction of these apps have been systematically evaluated and validated. Even fewer have been submitted for review by the Food and Drug Administration, which is tasked with monitoring use of some mobile technologies. Although there is tremendous potential for these apps to improve the health of individuals and communities, it is important that their use be driven by concrete data and demonstrated evidence of their efficacy, not solely by enthusiasm for sleek devices and apps with stylish user interfaces. To that end, it will be necessary to continue to monitor the progress of PoopMD as a tool to identify acholic stools. Version 2 of PoopMD, to be released in March, 2015, will allow for our team to *field test* the app, and assess whether the stool color analyzer provides accurate results to a larger sample of images. Furthermore, we will be able to better characterize typical use of the app, including how long users are logged into the app, the navigation pattern of the app including use of educational screens, and whether they use notifications and email their physicians regarding concerning stools. Finally, if parents enter the baby’s date of birth, version 2 will allow determination of the age at which a baby was found to have acholic stools in order to better understand the potential of PoopMD to significantly improve outcomes for children with BA.

## Supporting Information

S1 Table Supplemental raw data. (XLSX)

## References

[1] Sokol RJ, Mack C, Narkewicz MR, Karrer FM. Pathogenesis and outcome of biliary atresia: current concepts. J Pediatr Gastroenterol Nutr. 2003;37: 4–21 12827000 10.1097/00005176-200307000-00003

[2] Tiao M-M, Tsai S-S, Kuo H-W, Chen C-L, Yang C-Y. Epidemiological features of biliary atresia in Taiwan, a national study 1996–2003. J Gastroenterol Hepatol. 2008;23: 62–66. 10.1111/j.1440-1746.2007.05114.x 17725591

[3] Lien T-H, Chang M-H, Wu J-F, Chen H-L, Lee H-C, Chen A-C, et al Effects of the infant stool color card screening program on 5-year outcome of biliary atresia in Taiwan. Hepatol Baltim Md. 2011;53: 202–208. 10.1002/hep.24023 21140377

[4] De Vries W, de Langen ZJ, Groen H, Scheenstra R, Peeters PMJG, Hulscher JBF, et al Biliary atresia in the Netherlands: outcome of patients diagnosed between 1987 and 2008. J Pediatr. 2012;160: 638–644.e2. 10.1016/j.jpeds.2011.09.061 22082947

[5] Hung P-Y, Chen C-C, Chen W-J, Lai H-S, Hsu W-M, Lee P-H, et al Long-term prognosis of patients with biliary atresia: a 25 year summary. J Pediatr Gastroenterol Nutr. 2006;42: 190–195. 10.1097/01.mpg.0000189339.92891.64 16456414

[6] Schwarz KB, Haber BH, Philip R, Mack CL, Moore J, Bove K, et al Extra-hepatic anomalies in infants with biliary atresia: results of a large prospective North American multi-center study. Hepatology. 2013; 10.1002/hep.26512 PMC3844083 23703680

[7] Yoon PW, Bresee JS, Olney RS, James LM, Khoury MJ. Epidemiology of biliary atresia: a population-based study. Pediatrics. 1997;99: 376–382 9041292 10.1542/peds.99.3.376

[8] Chen S-M, Chang M-H, Du J-C, Lin C-C, Chen A-C, Lee H-C, et al Screening for biliary atresia by infant stool color card in Taiwan. Pediatrics. 2006;117: 1147–1154. 10.1542/peds.2005-1267 16585309

[9] Hsiao C-H, Chang M-H, Chen H-L, Lee H-C, Wu T-C, Lin C-C, et al Universal screening for biliary atresia using an infant stool color card in Taiwan. Hepatol Baltim Md. 2008;47: 1233–1240. 10.1002/hep.22182 18306391

[10] Smartphone Ownership 2013 | Pew Research Center’s Internet & American Life Project [Internet]. Available: http://pewinternet.org/Reports/2013/Smartphone-Ownership-2013.aspx. Accessed 2014 Feb 10

[11] Fox S, Duggan M. Mobile Health 2012. In: Pew Research Center’s Internet & American Life Project [Internet]. Available: http://www.pewinternet.org/2012/11/08/mobile-health-2012/. Accessed 2015 Feb 9

[12] Deeks JJ, Altman DG. Diagnostic tests 4: likelihood ratios. BMJ. 2004;329: 168–169. 10.1136/bmj.329.7458.168 15258077 PMC478236

[13] Mogul D, Zhou M, Intihar P, Schwarz K, Frick K. Cost-effective analysis of screening for biliary atresia with the stool color card. J Pediatr Gastroenterol Nutr. 2015;60: 91–98. 10.1097/MPG.0000000000000569 25221934

[14] Schreiber RA, Masucci L, Kaczorowski J, Collet J, Lutley P, Espinosa V, et al Home-based screening for biliary atresia using infant stool colour cards: A large-scale prospective cohort study and cost-effectiveness analysis. J Med Screen. 2014; 10.1177/0969141314542115 25009198

[15] Bakshi B, Sutcliffe A, Akindolie M, Vadamalayan B, John S, Arkley C, et al How reliably can paediatric professionals identify pale stool from cholestatic newborns? Arch Dis Child Fetal Neonatal Ed. 2012;97: F385–387. 10.1136/fetalneonatal-2010-209700 22933100

[16] Mary Meeker Code Conference Full Session Video [Internet]. Available: http://player.theplatform.com/p/PhfuRC/vNP4WUiQeJFa/select/BEqJpQcjAFeT?width=640&height=360. Accessed 2015 Feb 7

[17] Consumers use smartphones for 195 minutes per day, but spend only 25% of that time on communications [Internet]. Available: http://www.analysysmason.com/About-Us/News/Insight/consumers-smartphone-usage-May2014-RDMV0/. Accessed 2015 Feb 7

[18] WHO_PHP_34.pdf—WHO_PHP_34.pdf [Internet]. Available: http://whqlibdoc.who.int/php/WHO_PHP_34.pdf

[19] Pringle B, Colpe LJ, Blumberg SJ, Avila RM, Kogan MD. Diagnostic history and treatment of school-aged children with autism spectrum disorder and special health care needs. NCHS Data Brief. 2012; 1–8 23050521

[20] Wengrovitz AM, Brown MJ, Advisory Committee on Childhood Lead Poisoning, Division of Environmental and Emergency Health Services, National Center for Environmental Health, Centers for Disease Control and Prevention. Recommendations for blood lead screening of Medicaid-eligible children aged 1–5 years: an updated approach to targeting a group at high risk. MMWR Recomm Rep Morb Mortal Wkly Rep Recomm Rep Cent Dis Control. 2009;58: 1–11 19661858

[21] Integrated Public Alert and Warning System [Internet]. Wikipedia, the free encyclopedia. 2015. Available: http://en.wikipedia.org/w/index.php?title=Integrated_Public_Alert_and_Warning_System&oldid=628854391

[22] Steinhubl SR, Muse ED, Topol EJ. Can mobile health technologies transform health care? JAMA J Am Med Assoc. 2013;310: 2395–2396. 10.1001/jama.2013.281078 24158428

